# KRAS protein expression becomes progressively restricted during embryogenesis and in adulthood

**DOI:** 10.3389/fcell.2022.995013

**Published:** 2022-09-27

**Authors:** Marie-Albane Minati, Mohamad Assi, Maxime Libert, Sabine Cordi, Frédéric Lemaigre, Patrick Jacquemin

**Affiliations:** Université catholique de Louvain, de Duve Institute, Liver and Pancreas Differentiation Unit, Brussels, Belgium

**Keywords:** KRAS, development, epithelial cells, mesenchymal cells, mouse models

## Abstract

KRAS mutants are common in many cancers and wild-type KRAS is essential in development as its absence causes embryonic lethality. Despite this critical role in development and disease, the normal expression pattern of KRAS protein is still largely unknown at the tissue level due to the lack of valid antibodies. To address this issue, we used the *citrine-Kras* mouse model in which the Citrine-KRAS (Cit-K) fusion protein functions as a validated surrogate of endogenous KRAS protein that can be detected on tissue sections by immunolabeling with a GFP antibody. In the embryo, we found expression of KRAS protein in a wide range of organs and tissues. This expression tends to decrease near birth, mainly in mesenchymal cells. During transition to the adult stage, the dynamics of KRAS protein expression vary among organs and detection of KRAS becomes restricted to specific cell types. Furthermore, we found that steady state KRAS protein expression is detectable at the cell membrane and in the cytoplasm and that this subcellular partitioning differed among cell types. Our results reveal hitherto unanticipated dynamics in developmental, tissular, cell-specific and subcellular expression of KRAS protein. They provide insight into the reason why specific cell-types are sensitive to KRAS mutations during cancer initiation.

## 1 Introduction

The RAS family is composed of three genes, *Nras, Hras and Kras*, that encode four proteins called NRAS, HRAS and two splice isoforms of KRAS, namely KRAS4A and KRAS4B. RAS are small GTPases that cycle between “on/off” states with the help of guanine nucleotide exchange factors (GEFs) and GTPase-activating proteins (GAPs) which stimulate the active GTP-bound and inactive GDP-bound states, respectively (Barbacid, 1987; Lowy & Willumsen, 1993). The GTP-GDP exchange which regulates KRAS activity occurs at the plasma membrane (Stephen et al., 2014; Simanshu et al., 2017) where KRAS is anchored through prenylation. This membrane localization is essential for its activity (Cox et al., 2015) and results in the activation of downstream signaling pathways, such as the RAF-MEK-ERK signaling cascade (Simanshu et al., 2017). Trafficking of Kras between different cellular compartments has also been described as a process regulating its activity (Prior & Hancock, 2012). It should be noted that these studies are based on the use of tagged forms of KRAS which are overexpressed in cell lines and may behave differently from endogenous KRAS (Prior & Hancock, 2012).

The role of RAS in homeostasis, embryonic development, cancer, and genetic disorders gained the attention of researchers already in the eighties. Particularly, the role of *Ras* genes in embryonic development was first assessed using knockout mouse models. Mice lacking NRAS and HRAS showed normal viability and growth (Umanoff et al., 1995; Ise et al., 2000), while those lacking KRAS presented with a severe growth defect and died *in utero* around E15.5 (Korea et al., 1997; Johnson et al., 1997). Rescue experiments identified partial functional overlap between KRAS and HRAS or NRAS (Nakamura et al., 2008) Further analysis of *Kras* ^*-/-*^ embryos revealed that they displayed features of anemia, fetal liver defect (decellularization) (Johnson et al., 1997), motor neuronal defect (cell death) and ventricular myocardium atrophy (Korea et al., 1997). All these studies highlight KRAS as a master regulator of embryonic mouse development.

The expression pattern of the mouse *Kras* gene characterized by Northern blotting and quantitative real-time PCR revealed substantial variations in *Kras* expression among different organs and during development, from embryogenesis to adulthood (Leon et al., 1987; Newlaczyl et al., 2017). At the protein level, despite the importance of KRAS in health and disease, the lack of KRAS antibodies functional for tissue immunostaining prevented the characterization of KRAS protein expression and subcellular localization *in vivo* (Waters et al., 2017). Therefore, understanding RAS-driven developmental defects and tumor initiation remains complex.

To fill this gap of knowledge, we assessed KRAS protein expression at different stages of mouse embryonic development and in adults, using the *Kras* ^*Cit*^ mouse model, which has been recently developed by our lab (Assi et al., 2021). This model expresses a fusion protein called Citrine-KRAS (Cit-K), in which the fluorescent protein Citrine is fused in-frame with KRAS, and is transcribed under the control of the endogenous *Kras* promoter. Therefore, Cit-K faithfully reproduces the tissular expression pattern of KRAS protein, as well as its subcellular localization which can be assessed easily on tissue sections using a GFP antibody; importantly, Cit-K binds GTP and does not impact embryonic development, postnatal growth, and fertility of mice that homozygously express Cit-Kras (Kras^cit/cit^) (Assi et al., 2021). In the present paper, using this mouse model, we identified a dynamic organ- and cell-specific KRAS protein expression profile throughout mouse development and in adults.

## 2 Materials and methods

### 2.1 Mice

All procedures described in this study were performed with the approval of the animal welfare committee of the UCLouvain Health Sciences Sector (Brussels, Belgium; ethic number: 2021/UCL/MD/054).

*Kras* ^*Cit*^ mice expressing Cit-K have been described (Assi et al., 2021).

### 2.2 Embryo and organ collection

Embryos were collected at E13.5, E15.5 and E17.5 and were fixed in 4% paraformaldehyde at 4°C for 6–10 h before embedding in paraffin. Tissues dissected from 8-week-old mice were fixed in 4% paraformaldehyde at 4°C for 4–6 h before embedding in paraffin.

### 2.3 Immunofluorescent labeling

Six-μm tissue sections were deparaffinized and antigen retrieval was performed by heating the slides for 20 min in Tris-EDTA (pH 9) buffer in Lab Vision PT Module (Thermo Fisher Scientific). Sections were rinsed 5 min in phosphate-buffered saline (PBS), and permeabilized in PBS, 0.3% Triton X-100 (Sigma Aldrich) for 5 min at room temperature. After permeabilization, tissue sections were blocked in PBS, 3% low-fat milk, 10% bovine serum albumin, 0.3% Triton X-100 (blocking buffer) for 45 min at room temperature. Primary antibodies were diluted in blocking buffer and incubated with the samples overnight at 4°C. Primary antibodies and antibody dilution are listed in Table 1. Then, slides were washed with 0.1% Triton X-100 in PBS. Secondary antibodies were diluted in PBS, 10% bovine serum albumin, 0.3% Triton X-100, applied at 1/1000 dilution and incubated for 1 h at 37°C. Finally, sections were treated with the lipofuscin autofluorescence quencher (TrueBlack 23007, VWR) for 10 s. GFP labeling of *Kras* ^*+/+*^ tissue sections served as control to monitor the background level. Micrographs were taken with an Axiovert 200 fluorescent microscope (Zeiss) using the AxioVision software or with Cell Observer Spinning Disk Confocal Microscope using the ZEN software. Quantifications were performed on at least 3-5 fields per stage/organ using the FIJI (ImageJ) or ZEN softwares.

**TABLE 1:** Antibody used.

Antibodies	Origin	Dilution	Reference
α-actinin	Mouse	1/750	Sigma (A7811)
CD31	Rat	1/250	Dianova (D130)
E-CADHERIN	Mouse	1/1000	BD Biosciences (610182)
GFP	Goat	1/250	Abcam (ab6673)
HNF4	Mouse	1/350	R&D systems (PP-H1415-00)
NeuN	Mouse	1/100	Merck (MAB377)
SOX2	Rabbit	1/100	Abcam (ab97959)
SOX9	Rabbit	1/500	Home Made
VIMENTIN	Rabbit	1/100	Cell signaling (5741)

### 2.4 Analysis of KRAS subcellular localization

To study the subcellular localization of KRAS at the adult stage, immunofluorescence labeling for Cit-K, E-CADHERIN and DNA was performed on lung, intestine, pancreas and kidney sections of 8-week-old *Kras* ^*cit/cit*^ mice. Pictures of the sections were taken with the Cell Observer Spinning Disk Confocal Microscope using the ZEN software. To quantify the subcellular location of Cit-K, ZEN software was used: membrane *versus* cytoplasmic localization of Cit-K (green) was assessed semi-quantitatively by superimposing it to the signal intensity of E-CADHERIN (red; membrane) and DAPI (blue; nucleus), the area between E-CADHERIN and DAPI labeling being considered as cytoplasmic. Red, green and blue signal intensities were quantified along a line crossing the cell from one side to the other and passing through the nucleus. A discrete score of 0, 1, or 2 was assigned according to the signal intensity: 0 [0–50 arbitrary units (A.U.)], 1 (50–100 A.U.), and 2 (100–150 A.U). Quantifications were performed on 10 cells per cell type.

### 2.5 Western blotting

E15.5 embryonic or 8-week-old adult tissues from *Kras* ^*Cit/Cit*^ or *wild-type* mice were lysed and sonicated in lysis buffer composed of 50 mM Tris-Cl, 150 mM Sodium Chloride, 0.25% Sodium Deoxycholate, 1% NP-40, 1 mM Sodium Orthovanadate, 2% Sodium Dodecyl Sulfate (SDS) and containing Protease inhibitor (11836153001, Sigma Aldrich). Samples were kept on ice during the procedure. Then, samples were centrifuged (14000 g, 15 min, at 4°C) to pellet cell debris. Proteins were quantified using a Bradford assay. Samples containing 30 or 50 μg total proteins were separated on 12.5% SDS polyacrylamide gels. PVDF membranes (ISEQ00010, Millipore) were blocked with a solution of 5% low-fat milk diluted in Tris-buffered saline (TBS)/0.1% Tween-20 (Sigma-Aldrich) for 1 h at room temperature. Membranes were incubated overnight at 4 °C with primary antibodies against KRAS (own antibody) (Assi et al., 2020) or HSC70 (Santa Cruz sc-7298) (dilution 1:1000 for all antibodies). Then, membranes were washed with TBS/0.1% Tween-20 and incubated with secondary antibodies for 1 h at RT. After incubation, membranes were washed with TBS/, 0.1% Tween-20 and revealed using SuperSignal™ West Pico PLUS Chemiluminescent Substrate (34577, Thermo Fisher). HSC70 was used as loading control. Pictures were taken with the Fusion Solo S imaging system (Viber). Densitometry was measured using the FIJI software (ImageJ) and the ratio of the protein of interest over HSC70 was calculated.

### 2.6 Statistical analysis

Data were presented as means ± standard error of the mean (SEM). Statistical significance between two groups was calculated using unpaired Student’s t-test. For all statistical analyses, the level of significance was set at *p* < 0.05. Analyses were performed using GraphPad Prism software (version 9, GraphPad Software Inc., San Diego, CA, United States). *,*p* < 0.05; **,*p* < 0.01; ***,*p* < 0.001; ****,*p* < 0.0001; ns, not significant**.**

## 3 Results

### 3.1 KRAS protein is widely expressed during mouse embryogenesis

**FIGURE 1:**
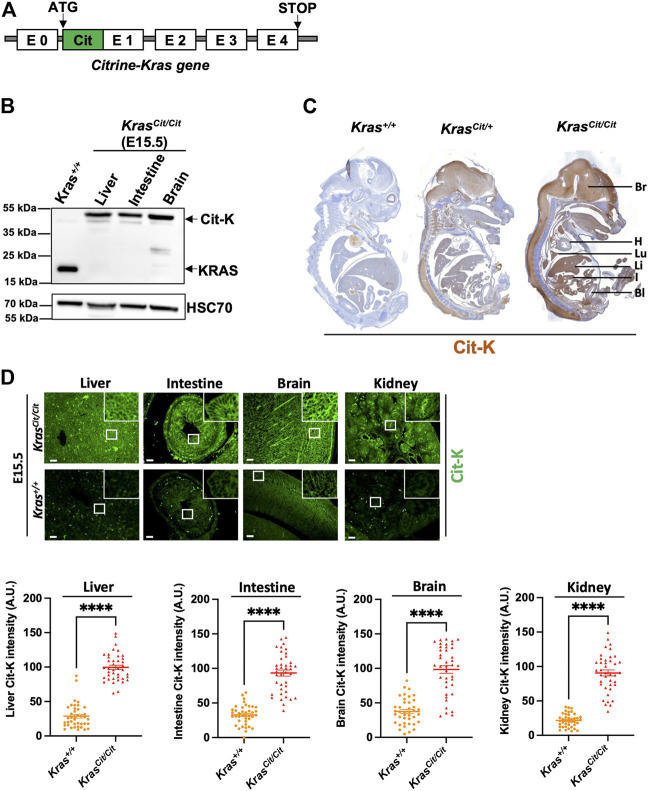
Citrine-KRAS (Cit-K) fusion protein is expressed in E15.5 *Kras* ^*Cit/Cit*^ embryos. **(A)** Construction of the *Kras* ^*Cit/Cit*^ mouse model generation using the CRISPR/Cas9 technology. **(B)** Western blot on liver, intestine, and brain protein lysates of E15.5 *Kras* ^*cit/cit*^ embryos. A KRAS antibody has been used to detect both KRAS and Cit-K. Heat shock cognate protein 70 (HSC70) has been used as loading control. W*ild-type* KRAS is detected to ∼22 kDa and Citrine-KRAS is detected to ∼48 kDa, as expected. The *Kras* ^*+/+*^ line corresponds to a protein extract from cells expressing *wild-type Kras* to show *wild-type* KRAS at the expected size of ∼22 kDa. **(C)** Immunohistochemistry using a GFP antibody (brown) on a whole section of E15.5 *Kras* ^*+/+*^, *Kras* ^*cit/+*^ and *Kras* ^*Cit/Cit*^ embryos counterstained with hematoxylin (blue). As expected, weak background was observed on the *Kras* ^*+/+*^ embryo, while the staining was most intense on the *Kras* ^*Cit/Cit*^ embryo. Bl, bladder; Br, brain; H, heart; I, intestine; Li, liver; Lu, lung. **(D)** Cit-K immunolabeling on E15.5 organ sections of *Kras* ^*+/+*^ and *Kras* ^*Cit/Cit*^ embryos. With the exception of intense non-specific labeling in tissue-resident red blood cells, no Cit-K labeling is observed in *Kras* ^*+/+*^ embryos. Graphs represent Cit-K intensity measured by FIJI software. One dot represents the mean of three measures of signal intensity on one cell. Data are represented as mean+/-SEM of 40 cells (*n* = 4 mice). Statistical significance was tested by Student’s *t*-test. ****, *p* < 0.0001. A.U. arbitrary unit.

To determine the expression pattern of KRAS, we used *Kras* ^*Cit*^ mice which produce a Citrine-KRAS (Cit-K) fusion protein under control of the endogenous *Kras* transcriptional regulatory elements (Assi et al., 2021) (Figure 1A). Therefore, detection of Cit-K on paraffin-embedded tissue sections with help of anti-GFP antibodies (citrine is an improved version of YFP) is a faithful substitute for detection of endogenous KRAS protein.

We generated homozygous *Kras* ^*cit/cit*^, thereby allowing Cit-K to be detected with the best efficiency. Western blot analyses on embryonic organs, including liver, intestine and brain revealed the presence of Cit-K at the expected molecular weight (Figure 1B). Immunohistochemistry on *Kras* ^*cit/cit*^ E15.5 embryos showed that most organs express Cit-K protein, whereas no specific signal was detected in *Kras* ^*+/+*^ controls (Figure 1C): a high level of expression was particularly seen in the brain, the lung, the liver, and the intestine. Notably, no/very weak expression was observed in the heart and the inner layer of the bladder. Immunofluorescent labeling of Cit-K on several organs confirmed the wide expression of KRAS (Figure 1D). The specificity of the immunostaining and immunofluorescent labeling in the *Kras* ^*cit/cit*^ embryos was confirmed by comparing it with the much lower level of staining and autofluorescence detected in *Kras* ^*+/+*^ embryos (Figures 1C,D). Since Cit-K serves as a surrogate for KRAS, these analyses document for the first time the broad expression profile of KRAS protein during embryonic mouse development.

### 3.2 KRAS protein expression gradually decreases in the mesenchymal compartment during mouse embryonic development

**FIGURE 2:**
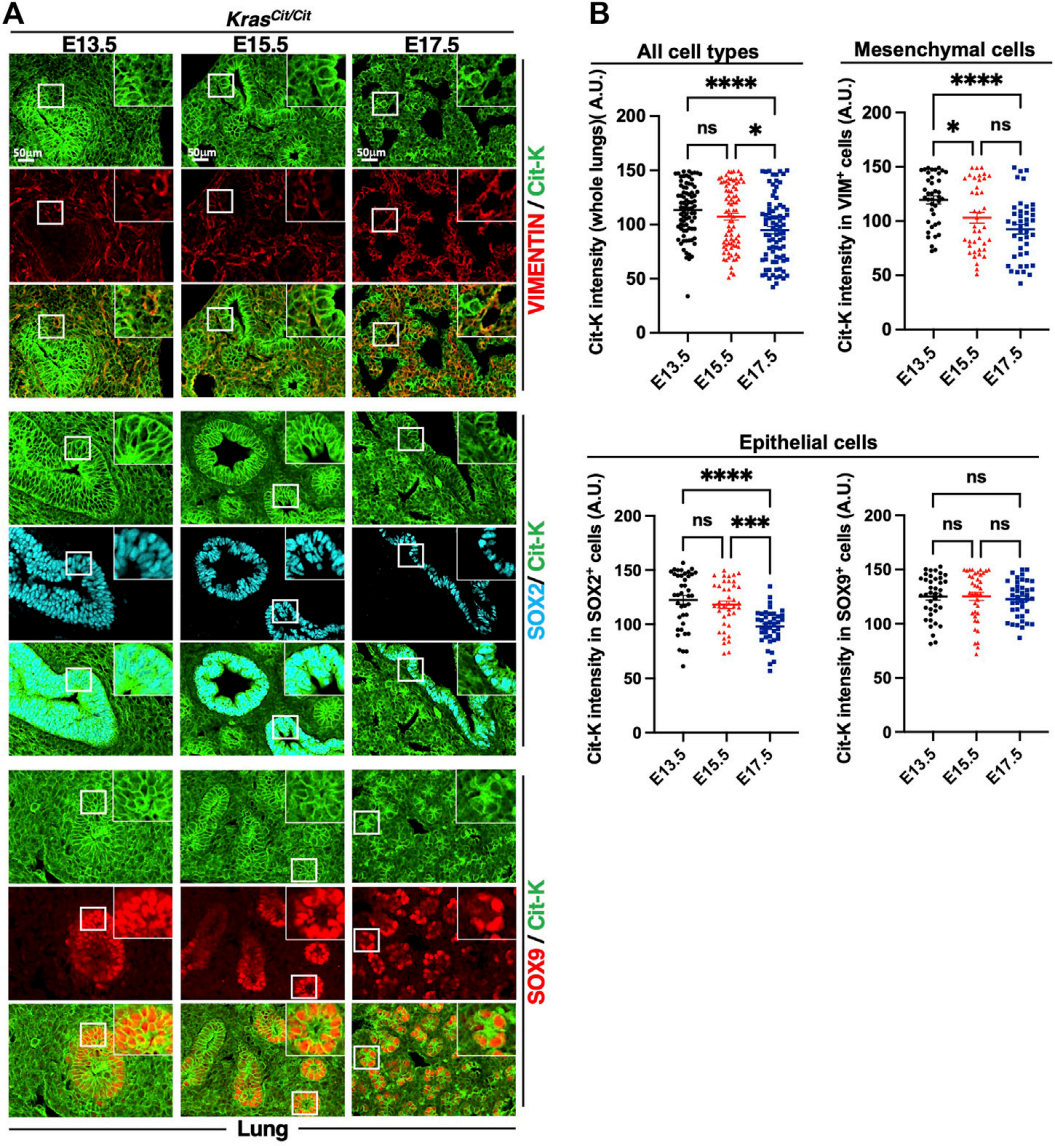
Cit-K is expressed in the developing lung. **(A)** Cit-K, VIMENTIN (VIM), SOX2 and SOX9 immunolabelling performed on lung sections of *Kras* ^*Cit/Cit*^ embryos at E13.5, E15.5 and E17.5. **(B)** Quantification by FIJI software of Cit-K signal intensity. One dot represents the mean of three measures of signal intensity of one cell. Data are represented as mean+/-SEM of 80 or 40 cells (*n* = 4 embryos). Statistical significance was tested by Student’s *t*-test. *, *p* < 0.05; ***, *p* < 0.001; ****, *p* < 0.0001; ns, not significant. A.U. arbitrary unit.

To study how the expression of KRAS evolves during embryonic development and cell differentiation, we first investigated Cit-K expression during lung development at E13.5, E15.5 and E17.5. By comparing these 3 stages, we observed a decrease of Cit-K expression over time (Figures 2A,B). This decrease mainly affected the lung mesenchyme: quantification of labeling obtained with anti-VIMENTIN and anti-E-CADHERIN antibodies showed that Cit-K expression remained constant in lung E-CADHERIN-positive epithelial cells (Supplementary Figure S1A,B), but significantly decreased in VIMENTIN-positive mesenchyme during progression of embryogenesis (Figures 2A,B).

The embryonic lung can be divided into a proximal and a distal portion, according to the expression of two SRY-box transcription factors, SOX2 and SOX9: SOX2 is found in the proximal airway epithelium whereas SOX9 is present in the distal lung epithelium (Hawkins et al., 2016). To investigate how KRAS expression evolved in the epithelium of both portions, we performed co-labeling of Cit-K and SOX factors. We observed that Cit-K expression decreased in the SOX2^+^ cells but stayed constant in SOX9^+^ cells (Figures 2A,B).

**FIGURE 3:**
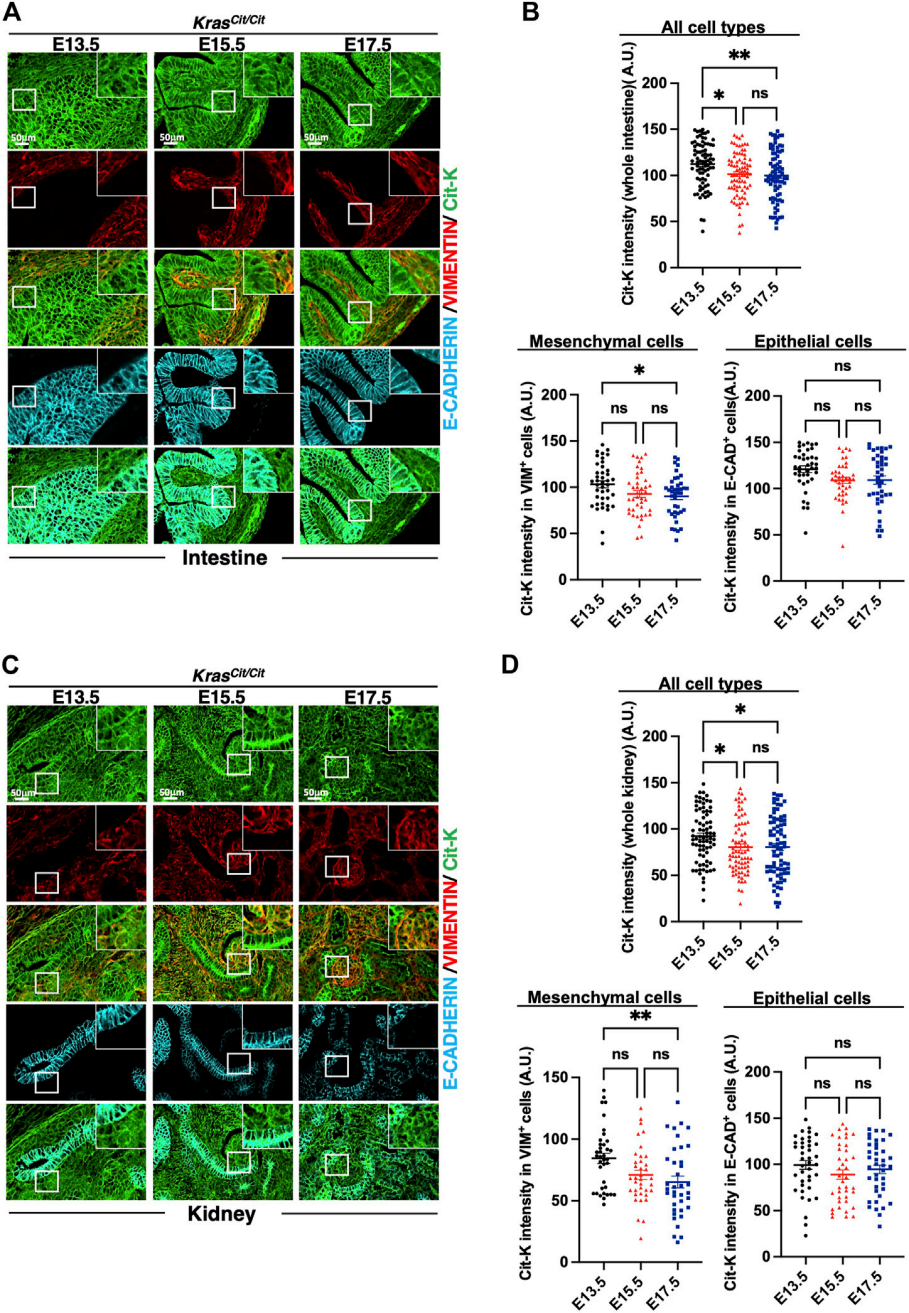
Cit-K expression is found in the developing intestine and kidney. **(A,C)** Immunolabeling for Cit-K, VIMENTIN (VIM) and E-CADHERIN (E-CAD) performed on embryonic intestine **(A)** and kidney **(C)** sections of *Kras* ^*Cit/Cit*^ embryos at E13.5, E15.5 and E17.5. **(B,D)** Quantification by FIJI software of Cit-K signal intensity in mesenchymal (VIM^+^) and epithelial (E-CAD^+^) cells of the embryonic intestine **(B)** and kidneys **(D)**. One dot represents the mean of three measures of signal intensity of one cell. Data are represented as mean+/-SEM of 80 (n = 4 embryos). Statistical significance was tested by Student’s *t*-test. *, *p* < 0.05; ns, not significant. A.U. arbitrary unit.

**FIGURE 4:**
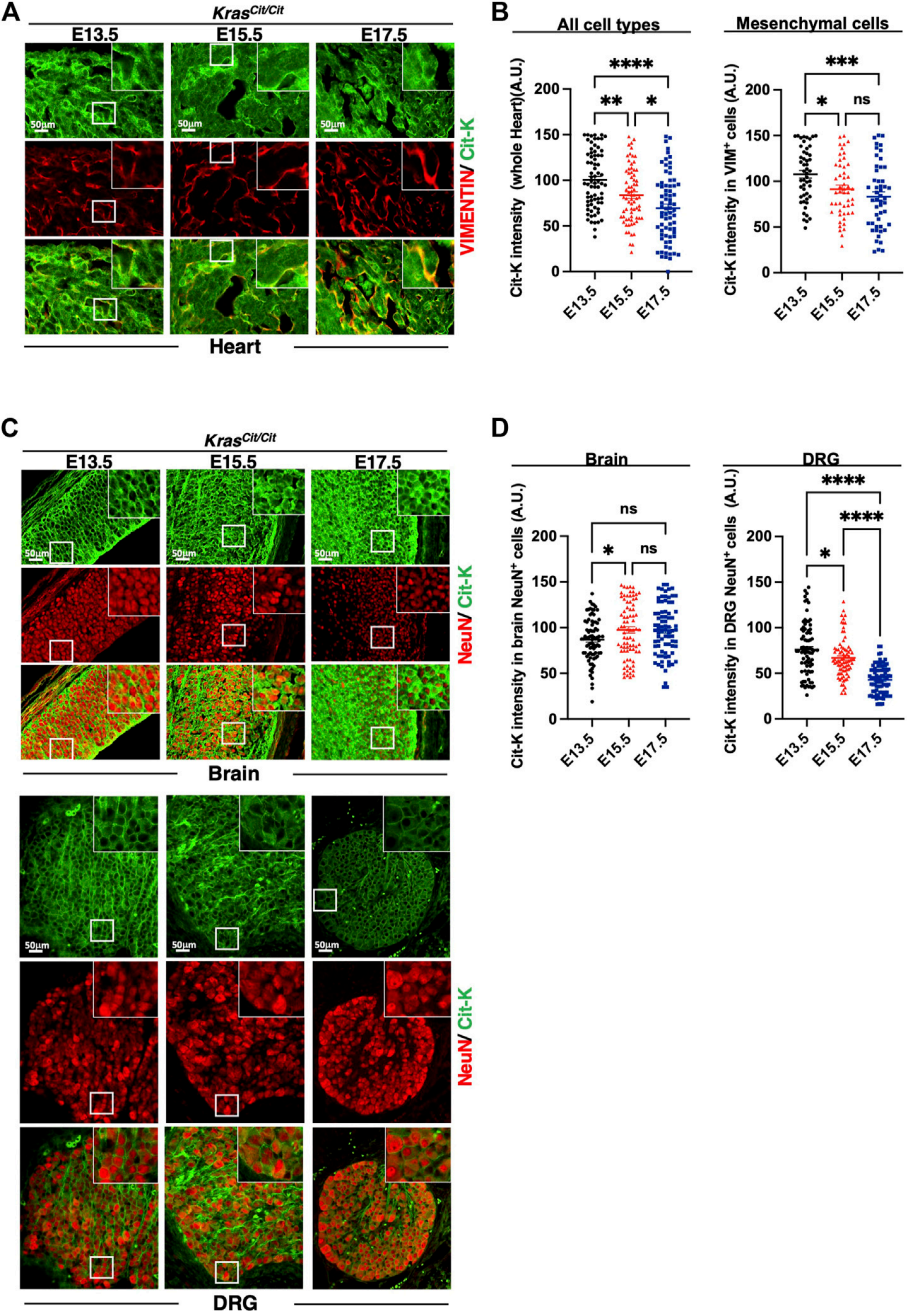
Cit-K expression is present in heart, dorsal root ganglion (DRG), and brain. **(A)** Immunolabeling for Cit-K and VIMENTIN (VIM) on heart sections of *Kras* ^*Cit/Cit*^ embryos at E13.5, E15.5 and E17.5. **(B)** Quantification by FIJI software of Cit-K signal intensity in whole heart tissue and mesenchymal (VIM^+^) cells. Data are represented as mean+/-SEM of 80 (n = 4 embryos). **(C)** Immunolabelling for Cit-K and NeuN (epithelial neuronal cell) on brain and dorsal root ganglion (DRG) sections of *Kras* ^*Cit/Cit*^ embryos at E13.5, E15.5 and E17.5. Comparison between DRG sections of *Kras* ^*+/+*^ and *Kras* ^*Cit/Cit*^ embryos (not shown) indicate that strong Cit-K labeling observed in nervous fibers is non-specific and can be explained by the auto-fluorescence of nervous fibers. **(D)** Quantification by FIJI software of Cit-K signal intensity in epithelial neuronal (NeuN^+^) cells. One dot represents the mean of three measures of signal intensity in one cell. Data are represented as mean+/-SEM of 80 cells (n = 4 embryos). Statistical significance was tested by Student’s *t*-test. *, *p* < 0.05; ***, *p* < 0.001; ****, *p* < 0.0001 and ns, not significant. A.U. arbitrary unit.

In intestine and kidney, progressive decrease of Cit-K expression was observed during embryogenesis, and, like in the lung, the decrease was predominant in the mesenchymal compartment (Figures 3A–D). Similar conclusions could be drawn from the analysis of the developing heart (Figures 4A,B). In contrast, in the nervous system, namely in the brain or dorsal root ganglia, the dynamics of Cit-K expression appeared different: brain neurons labeled for the marker NeuN showed increased Cit-K expression during development, whereas a decrease was detected in dorsal root ganglia (Figures 4C,D).

**FIGURE 5:**
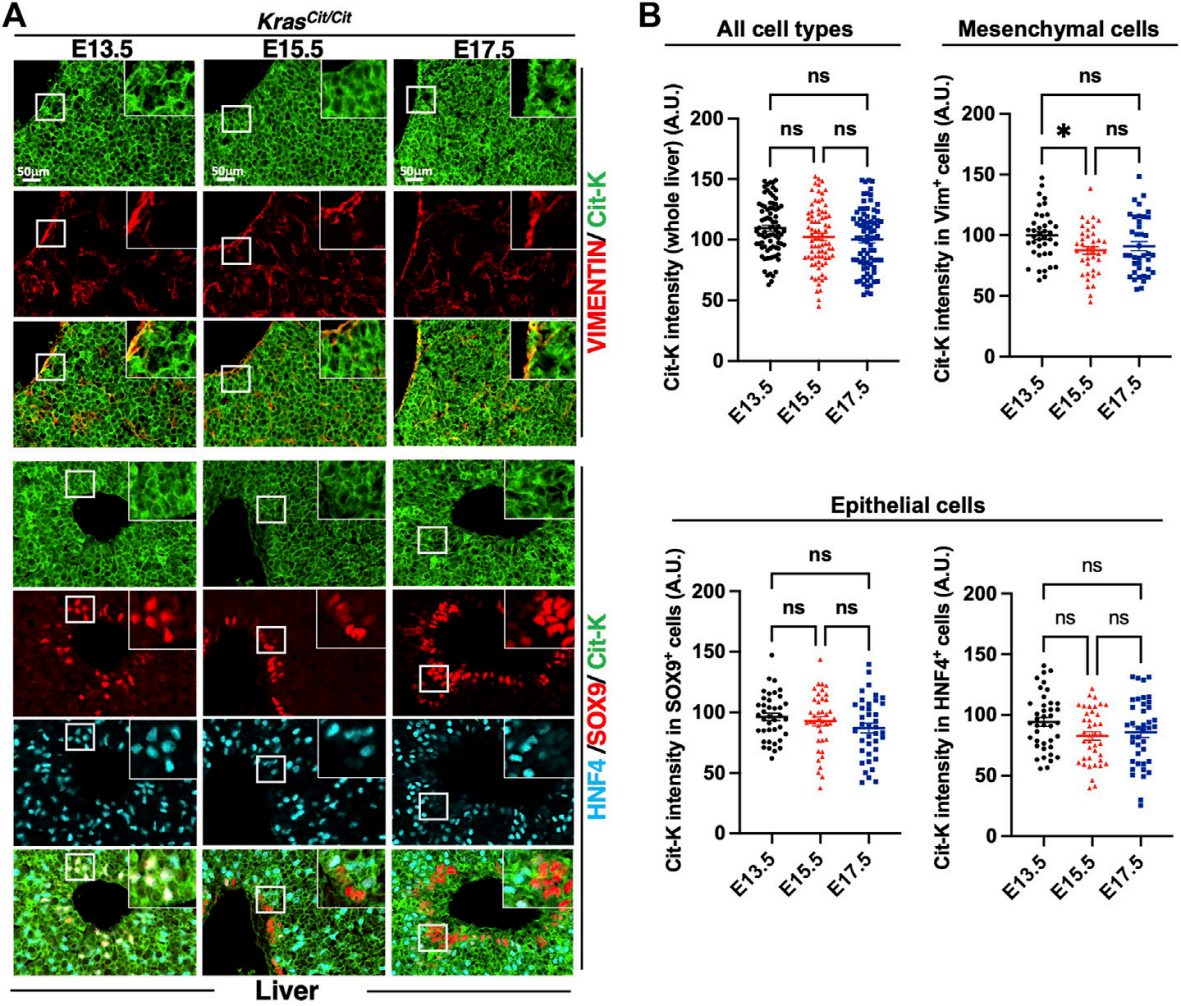
Cit-K is expressed in embryonic liver. **(A)** Immunolabeling for Cit-K, VIMENTIN (VIM), SRY-box transcription factor 9 (SOX9), and Hepatocyte nuclear factor 4 (HNF4) performed on liver sections of *Kras* ^*Cit/Cit*^ embryos at E13.5, E15.5 and E17.5. **(B)** Quantification by FIJI software of Cit-K signal intensity in the whole liver, mesenchymal (VIM^+^) cells, and two types of liver epithelial cells (SOX9^+^ or HNF4^+^ cells). One dot represents the mean of three measures of signal intensity in one cell. Data are represented as mean+/-SEM of 80 or 40 cells (n = 4 embryos). Statistical significance was tested by Student’s *t*-test. *, *p* < 0.05; ns, not significant. A.U. arbitrary unit.

Finally, we investigated KRAS expression in the embryonic liver. Cit-K expression showed a transient decrease in the mesenchyme between E13.5 and E15.5, but no additional reduction was detected at E17.5. In the epithelial compartment (Supplementary Figure S1C,D), Cit-K expression remained constant at all three stages tested in the SOX9^+^ (cholangiocytes) and HNF4^+^ (hepatoblasts and hepatocytes) cells (Figures 5A,B).

We concluded that KRAS protein expression in development is dynamic and tissue-specific, and that overall it is maintained in epithelial cells but tends to become progressively reduced in the mesenchymal compartment of several organs.

### 3.3 In adult tissues, KRAS protein expression is found in specific cell types

**FIGURE 6:**
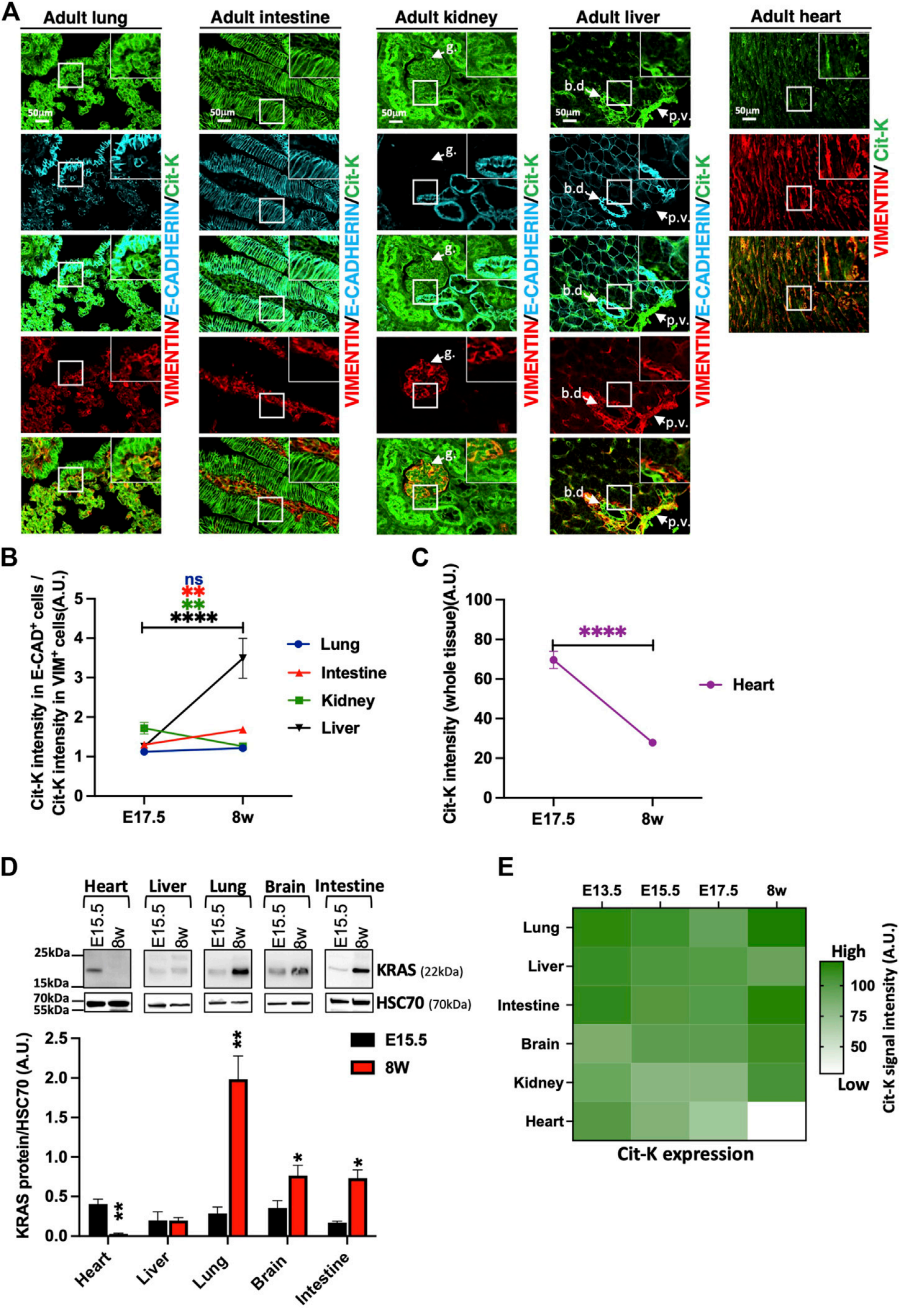
KRAS shows an organ- and cell type-specific expression pattern in adult mice. **(A)** Immunolabelling performed on lung, intestine, kidney, liver, and heart sections of 8-week-old *Kras* ^*Cit/Cit*^ mice for Cit-K, E-CADHERIN, as epithelial cell marker, and VIMENTIN (VIM), as mesenchymal cell marker. g, glomerulus; bd, bile duct; pv, portal vein. **(B)** Graph representing the ratio between the intensity of Cit-K labeling in epithelial E-CAD^+^ cells and that in mesenchymal VIM^+^ cells at E17.5 and the adult stage (8w, 8 weeks). Data are represented as mean+/-SEM of 40 cells (n = 4 mice). **(C)** Graph representing the Cit-K signal intensity (right) in the heart during embryogenesis and at the adult stage. Data are represented as mean+/-SEM of 40 cells (n = 4 mice). **(D)** Western blot analysis on total heart, liver, lung, brain and intestine lysate from E15.5 embryos (*n* = 4) and adult CD1 mice. A KRAS antibody has been used to detect KRAS. Heat shock cognate protein 70 (HSC70) has been used as loading control. Measure was performed by FIJI software, and statistical significance between E17.5 and 8w or E15.5 and 8w were tested by Student’s t-test. Data are represented as mean+/-SEM. *, *p* < 0.05; **, *p* < 0.01; ***, *p* < 0.001; ****, *p* < 0.0001; ns, not significant. A U. arbitrary unit. **(E)** Heatmap summarizing the Cit-K expression levels observed in the different organs at the different developmental stages indicated. The heatmap generated with Graphpad Prism was constructed from the mean levels of Cit-K expression measured in the different tissues at different times. A U. arbitrary unit.

Next, we studied how the pattern and levels of expression of KRAS evolved from the embryonic to the adult stage, and thus characterized Cit-K expression in several adult tissues and compared it with the embryonic period. We observed that Cit-K expression was maintained at a high level in E-CADHERIN^+^ epithelial cells of lung and intestine (Figure 6A). Cit-K expression in VIMENTIN^+^ mesenchymal cells of the intestine was lower after birth as compared to E17.5, resulting in a ratio of the intensity of epithelial Cit-K labeling on mesenchymal Cit-K labeling that increases over time (Figure 6A). In lung, this ratio remained unchanged between E17.5 and adult stage.

In kidney, this ratio decreased because of persistent high expression of Cit-K in the VIMENTIN^+^ glomerular cells and lowering expression in epithelia (Figures 6A,B). Conversely, the epithelial Cit-K/mesenchymal Cit-K ratio increased in liver between E17.5 and adults (Figures 6A,B); this increase resulted from stable Cit-K expression in the VIMENTIN^+^ compartment, essentially comprising the periportal mesenchyme, associated with rising Cit-K expression in the biliary epithelial cells. Importantly, Cit-K expression dropped after birth in hepatocytes, while Cit-K expression was observed in sinusoidal cells (Supplementary Figure S2A–C).

Cit-K expression in heart was in sharp contrast to that in other organs, because it decreased drastically after birth (Figures 6A,C); this result was also confirmed by Western blotting (Figure 6D). Low Cit-K expression remained detectable in VIMENTIN^+^ cells, while Cit-K was nearly not detected in the cardiomyocytes (Supplementary Figure S3A). Finally, Cit-K was absent in adult smooth and skeletal muscles (Supplementary Figure S3B and not shown). Taken together, our results reveal a highly dynamic and cell-type specific profile of KRAS protein expression which varies substantially from one organ to the other during growth from the prenatal to the adult period (Figure 6E).

### 3.4 KRAS protein expression is detected at the cell membrane and in the cytoplasm

**FIGURE 7:**
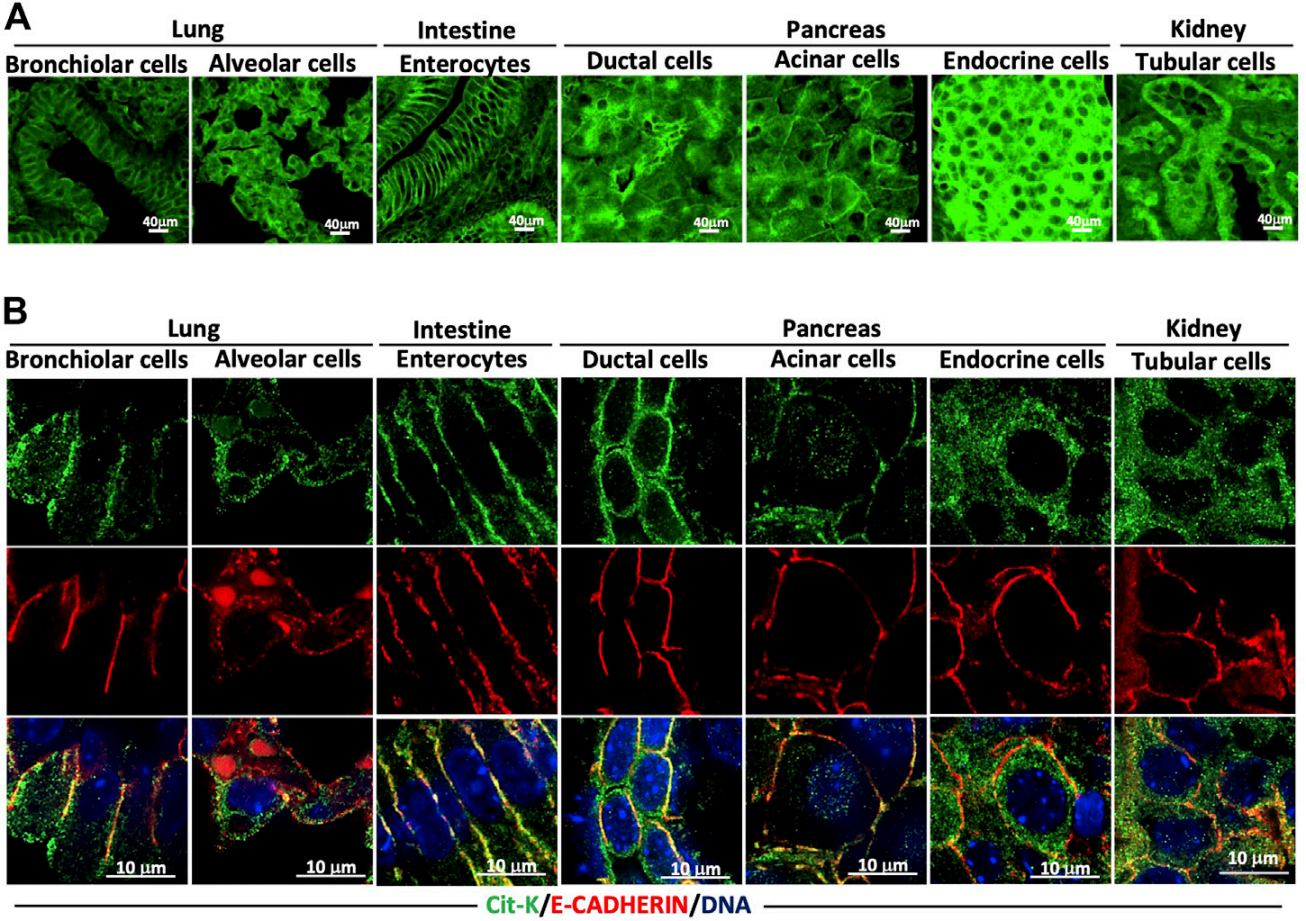
The cellular localization of KRAS varies according to the cell type. **(A)** Immunolabelling performed on lung, intestine, pancreas, and kidney sections of 8-week-old Kras^cit/cit^ mice for Cit-K. **(B)** Confocal images of Cit-K and E-CADHERIN (E-CAD; used as a cell membrane marker) immunolabelling performed on lung, intestine, pancreas, and kidney sections of 8-week-old Kras^cit/cit^ mice. DAPI was used to visualize cell nuclei.

Many *in vitro* studies have shown that KRAS has to be localized to the cell membrane to activate its downstream signaling pathways (reviewed in Haidar & Jacquemin, 2021). However, we found that the steady-state localization of Cit-K was predominant at the membrane in a number of tissues, and cytoplasmic in others. Our observations above (see for example the kidney in Figure 6A) suggest that this is not always the case *in vivo*. Low magnification pictures at first sight indicated that lung bronchiolar cells and intestinal enterocytes showed KRAS enrichment at the cell membrane, while alveolar cells, pancreatic endocrine cells and renal tubular cells showed the presence of significant amount of KRAS in their cytoplasm (Figure 7A). Indeed, confocal imaging of co-labeling with GFP, E-CADHERIN, and DAPI enabled us to subcellularly locate Cit-K and to semi-quantitatively assess its subcellular distribution, as described in Materials and Methods. This revealed that in the bronchiolar cells and enterocytes, Cit-K was preferentially present at the cell membrane, while in the alveolar, endocrine and tubular cells, Cit-K was equally partitioned between the membrane and the cytoplasm (Figure 7B). Double immunolabeling for Cit-K and for a marker of lysosomes, endoplasmic reticulum, and mitochondria indicated that Cit-K does not colocalize with these cellular organelles (Supplementary Figure S4).

## 4 Discussion

Since its discovery more than 40 years ago, multiple functions have been attributed to KRAS, including in cell proliferation, differentiation and apoptosis. From immunostaining studies performed shortly after its discovery (Chesa et al., 1987; Furth et al., 1987), it was assumed that KRAS was expressed ubiquitously; however, these studies have not been confirmed, and their conclusions were challenged when the capacity of available anti-KRAS antibodies to immunostain KRAS on tissues was questioned (Waters et al., 2017).

Previous KRAS expression data obtained by Northern or Western blotting came from bulk samples, preventing a clear idea of its expression pattern at the cell level. To address this issue in the absence of valid antibodies, we used the *Kras* ^*cit*^ mouse model which allows to detect the expression of KRAS using a GFP antibody (Assi et al., 2021). It should be noted that our *Kras* ^*Cit*^ mouse model detects both splicing isoforms of KRAS, KRAS4 and KRAS4B. As the *Kras* ^*Cit*^ fusion gene is located in the endogenous Kras locus, the resulting mRNA is expected to be spliced identically to endogenous Kras mRNA, yielding Cit-K4A and Cit-K4B proteins in an identical expression ratio to KRAS4A and KRAS4B proteins.

Our results show that KRAS is widely expressed in embryonic tissues. As embryogenesis progresses, its expression becomes tissue-restricted; this restriction is most prominent in the mesenchymal compartment, allowing us to conclude that KRAS expression is epithelial-enriched as birth approaches. After birth, this trend continues and is also accompanied by preferential expression in specific cell types. Low KRAS expression in the mesenchyme may explain why KRAS is weakly expressed in sarcomas and why *KRAS* mutations are rare in this type of cancer (AACR Project GENIE Consortium, 2017). From a functional point of view, it would be interesting to study whether the effectors downstream of KRAS are identical between epithelial and mesenchymal cell types. This could help understanding why KRAS gene amplification is associated with epithelial-to-mesenchymal transition and the degree of dedifferentiation in pancreatic cancer (Mueller et al., 2018).

A comparison of *Kras* mRNA (Leon et al., 1987) and Cit-K expression confirms the presence of *Kras* mRNA and KRAS protein in many organs. This comparison also confirms the absent or very low expression in skeletal muscle. In contrast, this study revealed significant expression of *Kras* mRNA in the heart. This difference compared to our study can be explained by the fact that we do not observe any expression in the cardiomyocytes, but that an expression is indeed detected in other cell types of the heart (e.g. in CD31^+^ cells) or by the presence of post-transcriptional regulatory mechanisms. The absence of KRAS expression in cardiomyocytes is surprising as a heart defect is probably the cause of the death in *Kras* ^*-/-*^ embryos (Korea et al., 1997). This can be explained either by an early expression of KRAS in the cardiomyocytes (before e13.5) or by the defect of another organ which affects the developing heart.

Interestingly, we observe KRAS expression in liver sinusoidal cells, a specialized endothelial cell type found notably in the organ. This observation may be linked to the discovery that *KRAS* mutations in these cells specifically lead to the development of hepatic vascular cavernomas (Janardhan et al., 2020), a benign tumor of the liver characterized by the presence of dilated endothelial-lined vascular spaces.

Our data show KRAS protein expression in several tissues. Muscle cells are a notable exception, regardless of their type (skeletal, cardiac, or smooth), as they exhibit low or undetectable KRAS expression. This suggests that the presence of a thin ventricular myocardium observed in *Kras* ^*-/-*^ embryos (Koera et al., 1997) results from a non-cell autonomous mechanism or from dysfunction of another organ that indirectly impacts cardiomyocyte function.

KRAS activity requires its location at the cell membrane, but our study reveals that KRAS can be observed in significant amounts in the cytoplasm in a subset of cell types. This is in sharp contrast with cultured cell lines where KRAS is essentially membrane-bound. Therefore, our *Kras* ^*Cit*^ mouse model represents an interesting tool to investigate the subcellular location of KRAS in tissues in a physiological or pathophysiological setting. In that context, the induction of enzymes that catalyze post-translational modifications of KRAS necessary for its membrane localization was shown to be highly context-dependent (Assi et al., 2020). Moreover, cytoplasmic expression of KRAS was associated with resistance of pancreatic acinar cells to tumor-promoting KRAS mutations: KRAS mutants were expressed, but not properly located for exerting their function (Assi et al., 2020). We therefore speculate that our present data showing KRAS predominantly in the cytoplasm of pancreatic endocrine cells provides an explanation to the insulin-producing β cells’ refractoriness to KRAS-mediated transformation (Gidekel Friedlander et al., 2009). A limitation of our study is the use of a fusion protein which could be processed differently and present a different trafficking from the endogenous form; still our validation of the Cit-K fusion protein (Assi et al., 2021) does not support this.

In conclusion, our results unravel the developmental, tissular, cell-specific and subcellular expression profile of KRAS protein, and provide insight into the mechanisms conferring sensitivity to KRAS mutations during tumor initiation. They also underscore the usefulness of *Kras* ^*Cit*^ mice, and suggest the development of similar approaches to investigate the biology of the other two members of the RAS family, NRAS and HRAS.

## Data Availability

The original contributions presented in the study are included in the article/Supplementary Material, further inquiries can be directed to the corresponding author.
