# Supplementary figures and images for: KRAS protein expression becomes progressively restricted during embryogenesis and in adulthood
Source: Front Cell Dev Biol. 2022 Sep 27;10:995013. doi: 10.3389/fcell.2022.995013 (PMC9551567; doi:10.3389/fcell.2022.995013)

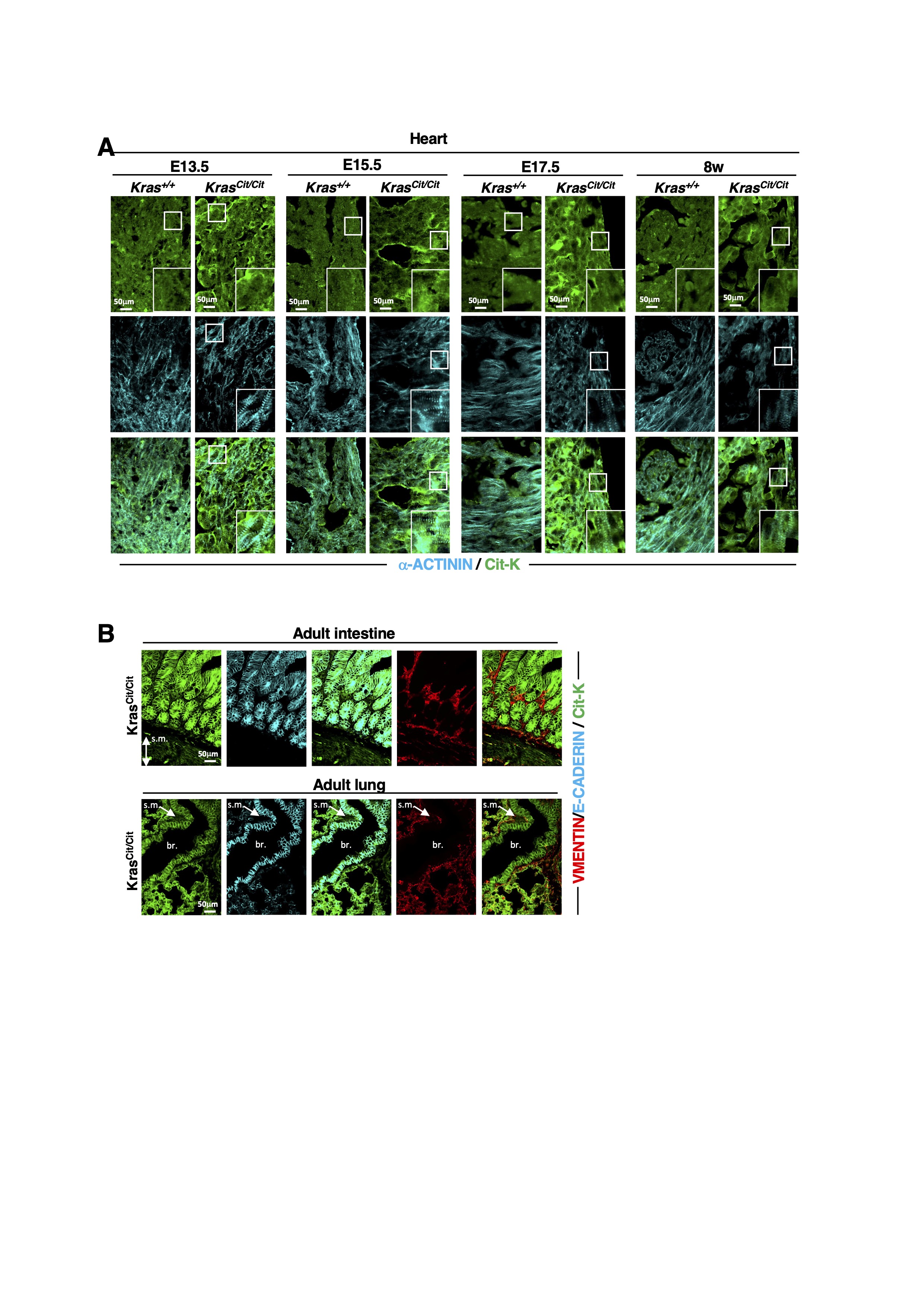

Supplement: Supplementary file 1 [file Image3.JPEG]

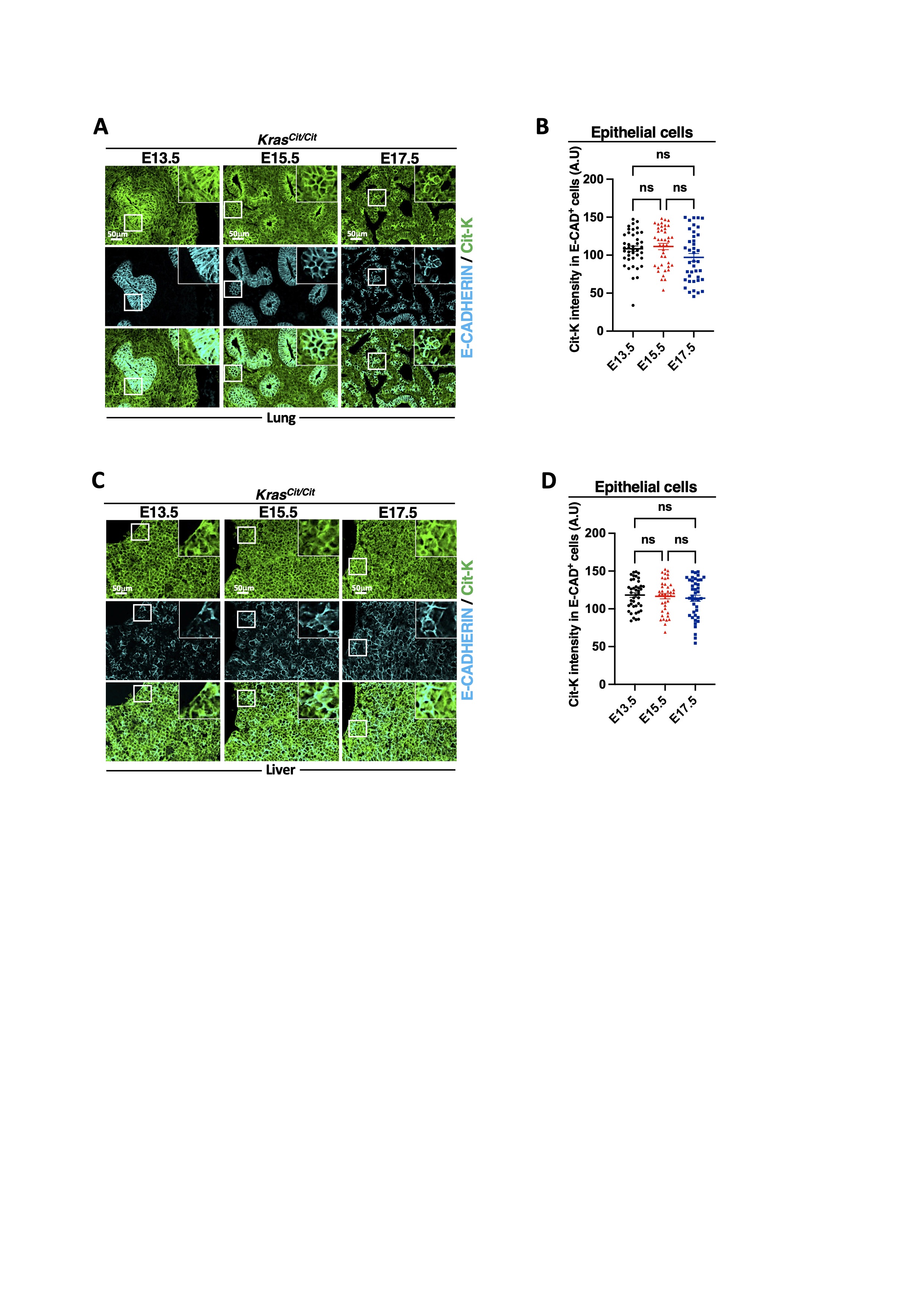

Supplement: Supplementary file 2 [file Image1.JPEG]

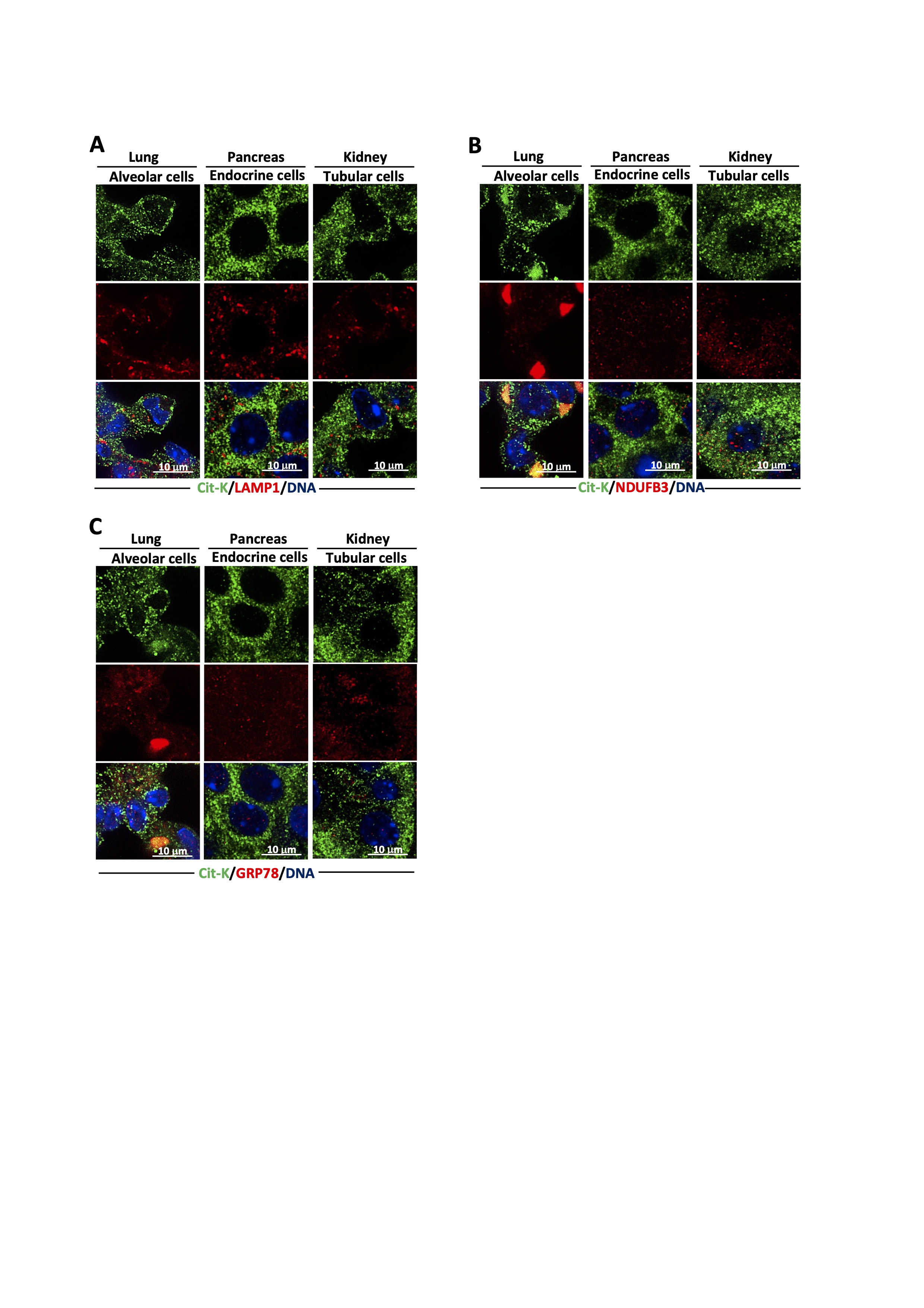

Supplement: Supplementary file 3 [file Image4.JPEG]

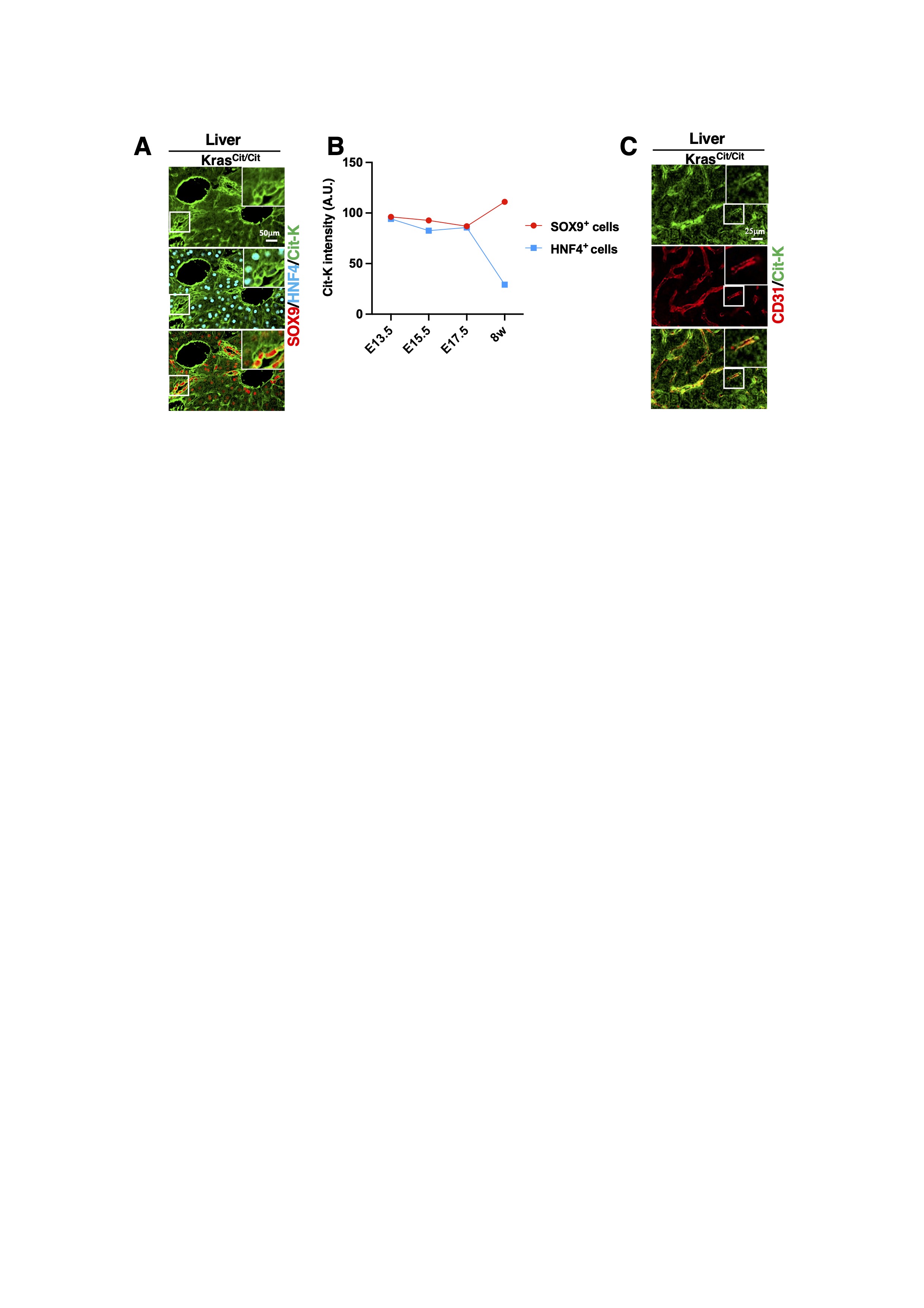

Supplement: Supplementary file 4 [file Image2.JPEG]
